# Development of a crowdsourcing- and gamification-based mobile application to collect epidemiological information and promote healthy lifestyles in Mexico

**DOI:** 10.1038/s41598-024-56761-4

**Published:** 2024-03-14

**Authors:** Kenny Mendoza, Víctor Eduardo Villalobos-Daniel, Alejandra Jáuregui, Isabel Valero-Morales, César Hernández-Alcaraz, Nelson Zacarías-Alejandro, Ricardo Omar Alarcon-Guevara, Simón Barquera

**Affiliations:** 1 Department of Nutrition, Harvard TH Chan School of Public Health, Boston, MA USA; 2 Center for Nutrition and Health Research (CINyS), National Institute of Public Health (INSP), Cuernavaca, Morelos Mexico; 3 Department of Noncommunicable Diseases and Mental Health, Pan American Health Organization, Washington, DC USA; 4 Queen Mary University of London, London, UK; 5 School of Public Health, National Institute of Public Health (INSP), Cuernavaca, Morelos Mexico

**Keywords:** Public health, Epidemiology

## Abstract

We developed a mobile application to promote healthy lifestyles and collect non-communicable disease (NCD) data in Mexico. Its theoretical foundations are supported by a framework-guided literature review. With design sprints, Scrum, Model-View-Controller, and Representational State Transfer architecture, we operationalized evidence-based nutrition/physical activity information into a crowdsourcing- and gamification-based application. The application was piloted for three months to monitor the response of 520 adults. Potential improvements were characterized, considering benchmarking, expert guidance, and standards. *Salud Activa* (English: *Active Health*) has two crowdsourcing modules: *Nutritional scanner*, scanning products' bar codes, providing nutritional data, and allowing new product registry feeding our databases; *Surveys*, comprising gradually-released NCD questions. Three intervention modules were generated: *Drinks diary*, a beverage assessment component to receive hydration recommendations; *Step counter*, monitoring users’ steps via Google Fit/Health—iOS; *Metabolic Avatar*, interconnecting modules and changing as a function of beverage and step records. The 3-month median of *Salud Activa* use was seven days (IQR = 3–12), up to 35% of participants completed a *Survey* section, and 157 food products were registered through *Nutritional scanner*. Better customization might benefit usability and user engagement. Quantitative and qualitative data will enhance *Salud Activa’s* design, user uptake, and efficacy in interventions delivered through this platform.

## Introduction

Multiple nations are now incentivizing research on eHealth, mHealth, and health-related mobile applications^1^. eHealth comprises the cost-effective and safe use of information and communication technologies (ICT) to support healthcare services, surveillance systems, and research and education on health concerns. mHealth is an eHealth subtype involving the use of smartphones or tablets to compile, store, and process data, as well as to conduct interventions through mobile applications^1^.

eHealth and mHealth interventions can enhance information accessibility and health awareness and trigger positive behavioral changes. Described as positive strategies for non-communicable diseases (NCD), they are functional to augment physical activity^2^, reduce sugar and fat intake ^3,4^, improve motivation among health program users^5^, and deliver nutritional counseling and diabetes education^6^. Regarding mobile application interventions, they have been linked to weight loss^7^, improvements in glycemic control^8^, and higher adherence to anti-hypertensive treatment^9^.

Besides interventions, monitoring the epidemiological dynamics of NCD is paramount in public health action evaluation against the epidemic. Crowdsourcing implemented through mHealth^10^ can facilitate gathering user information through mobile applications and allow the participating population to be "field investigators"^10^. As such, crowdsourcing becomes a pillar of participatory surveillance^11^, an emerging approach consisting of analyses of collected data, possibly through mobile applications, for subsequent utilization by the scientific community and decision-makers to improve health^11^.

Mobile applications must offer rewarding and beneficial experiences in exchange for information. The strategies must be tailored to the users' needs to augment and maintain engagement^12^. Using game elements in non-related game environments, known as gamification^13^, usually favors these goals. The use of gamification has been associated with turning potentially demotivating tasks into enjoyable experiences, providing various emotional and cognitive benefits during the knowledge acquisition process, and enhancing users’ satisfaction, self-esteem, and motivation^13–15^. Examples include avatars for self-representation and other rewards, through which gamification influences participation and motivation^13^. Furthermore, gamification can increase physical activity, improve mental health, and control NCD^13^.

To prevent potential effectiveness limitations of mHealth applications, the design process must integrate theories from human–computer interaction and behavioral and design sciences^16–18^. Documenting approaches to operationalize these field elements is essential for enabling future replication. As a hypothetical example, consider an application specifically designed to offer users feedback on their dietary habits: developers must delineate the behavior- and nutrition-related theoretical foundations underpinning content selection. Additionally, they must describe the design and technological components intended to ensure the attractive delivery of the message to the user. It is also relevant to garner evidence supporting different feedback strategies (e.g., pop-up messages on the application or alerts on the smartphone notification center) or the central gamification strategy.

Therefore, we aimed to describe the development process and pilot testing of a crowdsourcing- and gamification-based mobile application to promote healthy lifestyles and collect NCD-related epidemiological information in Mexico. The technological and theoretical design of the mobile application is described, delineating for the latter the standardized taxonomies of behavior change techniques and intervention taxonomies utilized to develop the intervention components of the application. In addition, we documented the lessons derived from this project and its areas of future improvement.

## Methods

Our team was composed of public health researchers, a quantitative behavioral designer, a graphic designer, a health communication specialist, and software developers. We aimed to develop an open-access smartphone application able to maintain user engagement and with the potential to promote healthy dietary and physical activity patterns through its use and implementation of intervention components. This project was developed in nine months through two stages. The first stage lasting six months, consisted of designing and programming the application. The second stage was a pilot to release the application to a sample of potential users to monitor their general response over three months. Subsequently, potential improvements for our application were described.

### Operational design

The application operation was designed based on two pillars. First was crowdsourcing, allowing us to collect information of interest from users, their environmental interactions, and lifestyle characteristics^10^. Second, gamification would help us create and maintain durable engagement of users^13^.

### Theoretical design for intervention components

The theoretical foundations of the application’s intervention components comprised standardized taxonomies of behavior change techniques, nutritional and physical activity interventions, and evidence-based information on diabetes prevention and control. To define the design space, we mapped four knowledge domains around diabetes that needed to be addressed to create a mHealth application: (1) pathophysiology and metabolism; (2) diet; (3) physical activity; and (4) behavior change. The identification of critical elements of these domains was performed through an extensive scientific literature review and a framework-guided approach (Table 1).

**Table 1:** Frameworks and evidence-based models guiding literature review and content selection for the development of a crowdsourcing- and gamification-based mHealth application for smartphones in Mexico.

Frameworks/model	Description	Selected target intervention component
Ominous octet^19^	The model describes a set of eight interconnected physiological abnormalities contributing to the development and progression of type 2 diabetes: muscle, liver, $\beta$-cell, adipocyte (accelerated lipolysis), gastrointestinal tract (incretin deficiency/resistance), $\alpha$-cell (hyperglucagonemia), kidney (increased glucose reabsorption), and brain (insulin resistance)^19^	To (1) alleviate stress on pancreatic beta cells and (2) mitigating ectopic fat accumulation
The Dietary Intervention Canvas (DIC)^20^	Guidelines for the development of evidence-based effective dietary interventions targeting diabetes control. It encompasses a blueprint to identify domains for diabetes care and map elements of eating patterns. It guides developers through six design processes^20^	(1) Reduction in the consumption of sugary drinks and (2) increase in the duration of moderate to vigorous physical activity
SLOTH model^21^	Economic and time-budget framework classifying the daytime into five domains (Sleep, Leisure, Occupation, Transportation, and Home) to identify feasible interventions to promote physical activity^21^	To encourage an increase in the number of steps throughout leisure, occupation, transportation times, and periods at home
The behavior change technique (BCT) taxonomy v1^22,23^	A comprehensive taxonomy consisting of 93 behavior change techniques and categorized into 16 groups^22,23^	Education regarding the effects and onset time of the exposure (sugary drinks intake and step count) and outcome (diabetes)

To map diabetes pathophysiology and metabolism, the Ominous Octet model involving a set of biological causes leading to this condition was selected^19^. The Dietary Intervention Canvas (DIC) developed by the behavioral designer co-leading this project (V.-D.V.E.)^20^ was utilized to identify and characterize (“mapping”) potential dietary modification strategies. Integrating principles from design, behavioral, and nutritional sciences, DIC is a translational research-based culturally- and life course-sensitive framework constructed to guide the development of evidence-based effective dietary interventions targeting diabetes control^20^. DIC consists of a blueprint designed to identify the following domains for diabetes care: causal mechanism, medical nutrition therapy specifications, target population, value proposition (any dietary intervention should derive in net positive value to the target population). It maps elements of eating patterns: obtaining (selecting and acquiring food items), processing (preparing and cooking), ingesting, post-ingesting, and fasting stages. DIC guides developers through six design processes: (1) to understand the causal model of the health outcome (i.e., diabetes); (2) to observe the context, population, and constraints; (3) to generate, nurture, and examine ideas to address the causal pathway; (4) to build (transforming ideas into prototypes); (5) to test prototypes; and (6) iteration to refine prototypes progressively and systematically^20^. Potentially effective intervention elements to enhance physical activity were identified by using the Sleep, Leisure, Occupation, Transportation, and Home-based activities (SLOTH) model^21^. Briefly, the SLOTH model is an economic and time-budget framework classifying the daytime into five domains: Sleep, Leisure, Occupation, Transportation, and Home. It enables to identify feasible interventions by considering economic factors to promote physical activity within each domain (excluding sleep)^21^. Lastly, behavioral change techniques were mapped from a taxonomy encompassing 93 approaches selected by expert consensus and that can be integrated in designing interventions^22,23^.

### Integration of design elements

The theoretical foundations and the elements of the operational pillars (i.e., crowdsourcing and gamification) were integrated through a modified version of Design Sprint^24^, a framework that considers the user experience in software development. Briefly, this five-stage process consisted of: (1) objectives definition and theoretical exploration with experts; (2) generation and selection of ideas and concepts formation; (3) construction and evaluation of prototypes; (4) iteration and re-evaluation of concepts; (5) benchmarking and final specifications of the concepts developed. The selected content was inserted into the application in Spanish.

### Technological development

Scrum^25^ guided operational activities, an agile work methodology for developing complex projects. This method allows workgroups to insert a new product into the corresponding markets in relatively short periods; it is an iterative process, and the current project consisted of one-month six sprints developed in four events. First, sprint planning, which involved meetings to define objectives and tasks. Second, short daily meetings of approximately 15 min to monitor work progress and adapt tasks and activities. Third, sprint reviews with meetings at the end of each sprint to show the progress of the developed product, its functionality, and elements to add in the next sprint. Fourth, sprint retrospective, a meeting to assess the implementation of Scrum in the last sprint.

Four programmers developed the application software using the following tools. Git is a management software to control application versions with a relevant number of source code files. Given its compatibility with several programming languages, Microsoft's Visual Studio Code (VSCode) was used as a source code editor. Bitbucket, a web-based code repository hosting service (by Atlassian) for application version control, is helpful for projects using Mercurial/Git revision control systems. Nodemon is a command-line tool that restarts the local development server when a source code change is detected.

The front end (client) was developed through Model-View-Controller (MVC) architecture, a model that separates data, logic, and user interfaces and meets design pattern objectives. The Model component makes requests to the databases to send or receive information. The databases were hosted on the application and MySQL (open-source management system). The View component is the code that presents the data, and the Controller component links the view and the model. The backend (server) was developed through Representational State Transfer (REST) architecture for network applications. REST is an interface between systems using the Hypertext Transfer Protocol (HTTP) to obtain/perform operations on those data. The transmission and exchange of information were developed using JavaScript Object Notation.

Tableau Software was used to host and display digestible data for "non-programmers." This software enables the analysis of the number of registered users and their general characteristics, as well as obtaining the number of log-ins and the percentage of completion of tasks programmed on the application components.

The application was temporarily inserted into Play Store (Android) and App Store (iOS) by creating the corresponding developer accounts.

### Pilot stage: application release to potential users

The pilot stage aimed to monitor the general use and response to the application from a sample of potential users. The study sample comprised students, teachers, and administrative personnel from the Universidad Autónoma del Estado de Morelos (Spanish acronym: UAEM). Recruitment of participants was facilitated by official academic agreements established between UAEM and the Mexican National Institute of Public Health (Spanish acronym: INSP). Individuals identified to be in a stage of contemplation or preparation concerning modifications to their lifestyle, changes in dietary patterns (e.g., reduction in sugar-sweetened beverage intake), and physical activity levels were included. Spanish questionnaires, validated and adapted from the Transtheoretical Model, were utilized to assess the stage of behavior change regarding physical exercise and dietary patterns among participants^26,27^. These selection criteria around individuals in a stage of contemplation or preparation emerged from evidence suggesting relevant engagement and health benefits in participants at these behavioral change stages using mHealth platforms promoting healthy dietary and physical activity behaviors to prevent and control NCD^28–30^. Participants who did not have a smartphone were not approached for invitation. However, according to national data, a widespread use of smartphones among adults in Mexico was observed by the recruitment period (2019: 94.6% among cellphone)^31^.

Invitations and study recruitment were carried out between August 12 and September 12, 2019, in UAEM's facilities through direct in-person contact or digitally through electronic flyers. Bachelor’s degree in nutrition and dietetics students trained explicitly for this project comprehensively explained study objectives and procedures to participants. Once they consented to participation, they administered electronic questionnaires through Redcap to collect information on sociodemographic characteristics and evaluate stages of behavior change. When meeting eligibility criteria, participants were instructed to download our application, create a user account, and they were assigned a personal identification number. In addition, participants were asked to update or download Google Fit (Android users) or Health (iOS users) in case of not having them, as these applications are needed for the operation of one of the components of our application.

The pilot stage lasted three months. During this period, daily users' application log-ins were assessed. Additionally, users' interaction with two application components was monitored: (1) The percentage of questions answered by users embedded in a crowdsourcing-based module containing several short questionaries and (2) the use of another crowdsourcing component enabling users to interactively register food products to our databases and obtain nutritional recommendations in return. The components mentioned above are described in detail in subsequent sections of this document. The piloting stage was finalized on December 12, 2019. All participants received a letter of appreciation in response to their participation. Those who interacted with the application for 30 or more consecutive days received a thermally insulated water bottle as a reward after project termination. Research indicates that providing individuals with insulated bottles has been linked to fostering healthier practices related to water consumption^32^.

### Identifying areas of improvement

Subsequently, opportunities to improve design and technological components were identified. This process involved further benchmarking and feedback from a mobile application development expert (ROAG). Current standards for health-related mobile applications^33^ were considered, comprising criteria that can help stakeholders to improve usability, privacy, security, appropriateness and suitability, transparency and content, safety, technical support and updates, and technology^33^.

### Ethics declarations

According to national and international regulations, our protocol adhered to the STROBE guidelines and this project was approved by the Ethics and Research Committees of the Mexican National Institute of Public Health (INSP) (approval number: 1550), in agreement with The Mexican Ministry of Health and the Declaration of Helsinki. Following ethical standards for research, a privacy notice was provided to all participants, and they electronically signed informed consent via our application.

The protection of personal information inserted into our application was consistent with Mexican laws. We implemented data encryption, a firewall and antivirus, and constant software and hardware maintenance. Data were encrypted end-to-end to prevent third-party access. Personal verification processes with the confirmation of codes through users' mobile phone numbers or email were employed. The technical, scientific, and administrative staff signed confidentiality letters and data custody to protect information.

## Results

**Figure 1:**
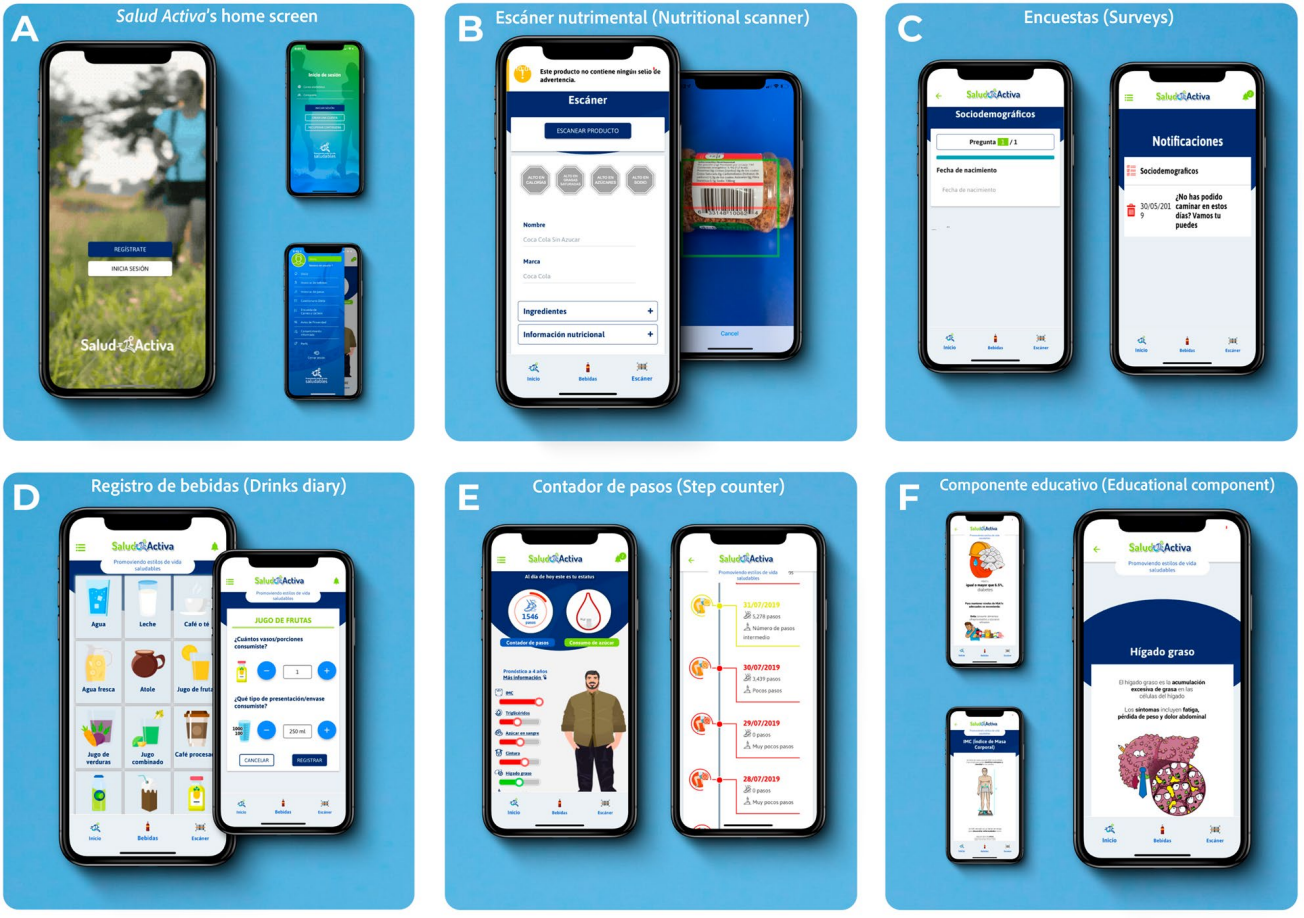
The original design of *Salud Activa* (translation into English: Active Health): A crowdsourcing- and gamification-based mobile application. (**A**) Displays *Salud Activa’*s home screen. (**B**) Displays module 1, named *Escáner nutrimental* (Nutritional scanner), a component that scans processed products' bar codes via smartphone camera to provide feedback to the user on the nutritional composition of registered products displaying the following messages: "High in calories, sugars, saturated and trans fats, or sodium." This module allows users to register new products to feed our databases for subsequent validation and use for research purposes. (**C**) Displays module 2, named *Encuestas* (Surveys), which comprises sets of non-communicable disease-related questions gradually released over time to prevent excessive burden in users. (**D**) Displays module 3, an intervention component named *Registro de bebidas* (Drinks diary). Users register beverage intake in this interactive semi-quantitative dietary assessment component linked to Mexican food composition tables to estimate the amount of sugar in these products. As a reward, hydration recommendations are given, graphically represented through a circle that changes in color depending on the record: red when sugar consumption exceeds 50 g/day, yellow when 25–50 g/day, and green when less than 25 g/day. (**E**) Displays module 4, another intervention component named *Contador de pasos* (Step counter). Synchronized with Google Fit (Android) or Health (iOS), it monitors user steps number and provides feedback on physical activity as the interaction progresses. Participants are notified based on their daily step count: low (< 3000 steps), minimal (3000–4999 steps), average (5000–7999 steps), good (8000–10,000 steps), and excellent (> 10,000 steps). (**F**) Displays a complementary module offering users information on non-communicable diseases, named *Componente educativo* (Educational component).

The design phase suggested a modular, interconnected concept. Six modules were created: two based on crowdsourcing and three combining gamification and crowdsourcing. An educational component was also inserted. Our mobile application was named *Salud Activa* (translation into English: *Active Health*). The application's initial interface comprises a welcoming screen for the user's registration, log-in, and a brief description of *Salud Activa*'s components. The home screen includes a lower bar with a start button displaying the main navigation bar and two buttons for quick access to the main modules (Fig. 1A).

### Crowdsourcing modules

Module 1: *Escáner nutrimental* (English: *Nutritional scanner*; Fig. 1B). By utilizing information from a commercial data set of ≈ 5000 foods and beverages in the Mexican market, the module recognizes and offers guidance on the nutritional composition of these products. This component scans products' bar codes by accessing the smartphone camera to display the following messages in black-and-white octagons based on the Pan American Health Organization nutrient profile model^34^: "High in calories, sugars, saturated and trans fats, or sodium." Additionally, *Escáner nutrimental* allows users to register new products to feed our databases, for which participants must take three photographs of a given product (front, nutritional composition table, and list of ingredients). Validation of this information is carried out using a digital management platform.

Module 2: *Encuestas* (English: Surveys; Fig. 1C). This module is composed of question sets taken from Mexican National Survey System questionnaires focused on sociodemographic characteristics, self-reported medical diagnoses of diabetes and cardiovascular disease, information on risk factors (i.e., physical activity, sugary drink intake, sedentarism, blood pressure, glycemia, dyslipidemia, obesity, body image, and depression), and knowledge on nutrition labeling. Each question was scheduled to be gradually released over 85 days following users' registration to prevent burden. Pop-up notifications reminding the release of questions were programmed every time participants logged into *Salud Activa*.

### Description of intervention components

Module 3: *Registro de bebidas* (English: Drinks diary; Fig. 1D). This module has iconography for each drink and an interactive menu where users can register the intake of beverages by type, number of portions, and size. *Salud Activa* displays the amount of sugar consumed in processed beverages (e.g., soft drinks or industrialized juices) and that added in homemade preparations (e.g., tea or coffee). This component is linked to Mexican food composition tables to estimate the amount of sugar in these products. The nutritional feedback *Registro de bebidas* provides is graphically represented through a circle that changes in color depending on the record: red when sugar consumption exceeds 50 g/day, yellow when 25-50 g/day, and green when less than 25 g/day^35^. This information is derived from the World Health Organization's (WHO) recommendations, which advocate for strong (10% of total energy intake = 50 g/day) and conditional (5% of total energy intake = 25 g/day) limits on sugar intake for adults and children following a typical 2000-cal diet^35^. *Registro de bebidas* can also be used as a semi-quantitative tool to assess beverage consumption after data processing.

Module 4: *Contador de pasos* (English: Step counter; Fig. 1E), which is synchronized with Google Fit (Android) or Health (iOS). This module allows users to track daily steps and visualize step history. Considering such information, participants receive feedback on physical activity levels as their interaction with the application progresses: low (< 3000 steps), minimal (3000–4999 steps), average (5000–7999 steps), good (8000–10,000 steps), and excellent (> 10,000 steps). For the correct functioning of this component, users must open the application at the beginning and end of the day before walking and sleeping, respectively.

**Figure 2:**
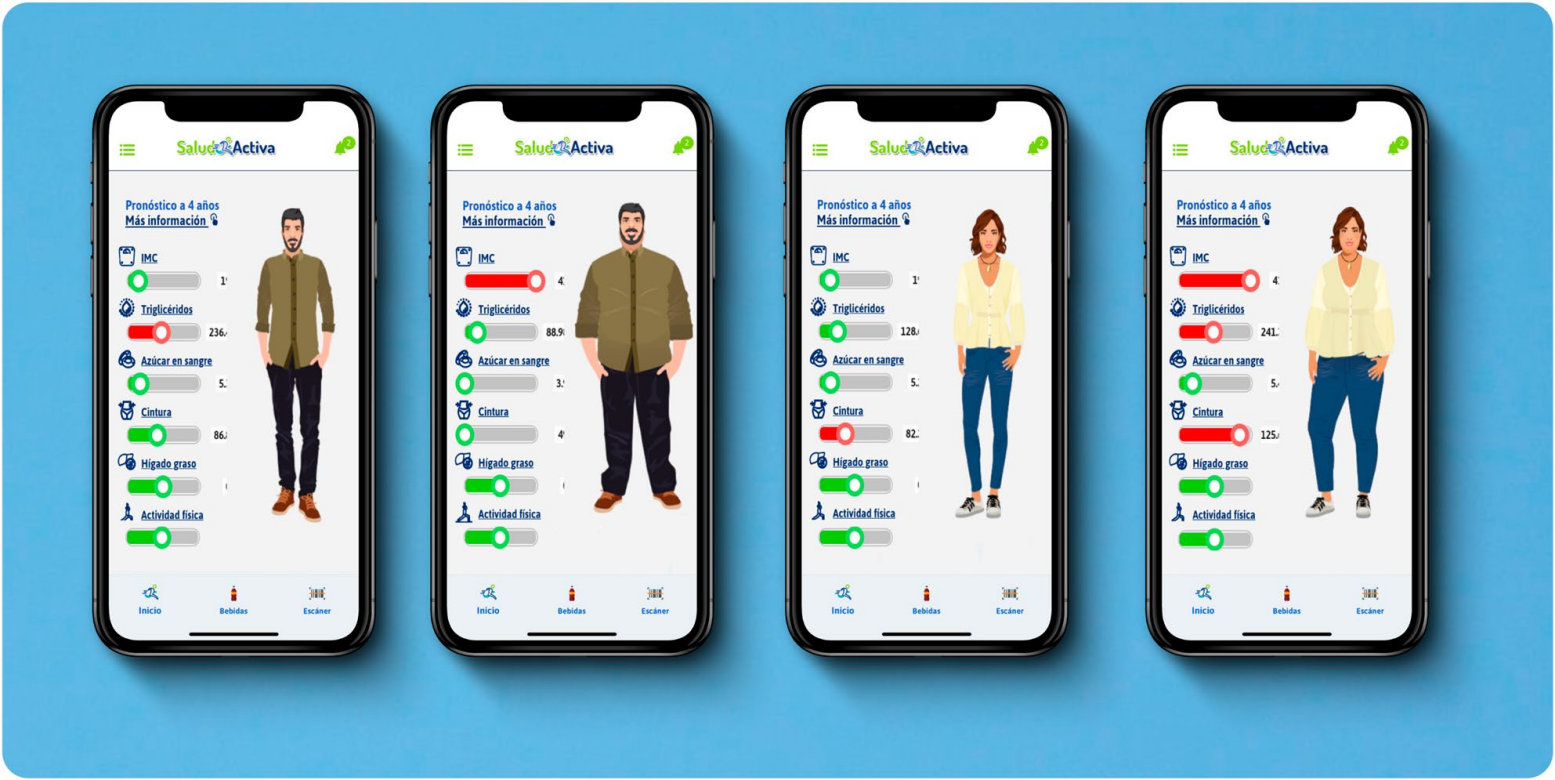
*Avatar Metabólico* (translation into English: Metabolic Avatar): the principal gamification strategy and the hub node establishing interconnection with *Salud Activa*’s components. *Avatar Metabólico* (module 5) is a self-representation of users functioning as an intervention component. *Avatar Metabólico* displays values of the following indicators as bars: (1) body mass index; (2) triglycerides; (3) hemoglobin A1c; (4) waist circumference; (5) fatty liver; (6) physical activity. Unhealthy levels are in orange or red and optimal levels in green. The indicators and body image of *Avatar Metabólico* change as a function of the record of steps and drinks. The higher the sugary drinks registered/consumed and the lower the number of steps, the higher the indicator levels, and vice versa. *Avatar Metabólico* has four body image patterns: (1) normal weight; (2) overweigh; (3) obesity; (4) morbid obesity.

Module 5: *Avatar Metabólico* (English: Metabolic Avatar). This element is the principal gamification strategy to generate engagement and the "hub node" establishing interconnection with and between the previous components (Fig. 2).

*Avatar Metabólico* is a self-representation of users launched after participants register their weight and height. The application calculates participants' body mass index (BMI) with these data. Then, it displays initial values of NCD indicators in the form of bars: (1) triglycerides; (2) hemoglobin A1c (called “blood sugar”); (3) waist circumference; (4) fatty liver; (5) physical activity. These markers are shown in a traffic light-style bar and are based on healthy/control cut-off points proposed by WHO: Unhealthy levels are shown in orange or red; optimal levels in green. The indicators and body image of *Avatar Metabólico* change as a function of the record of steps and drinks. Baseline marker values and their nature of change were established using linear predictive equations based on longitudinal data (baseline: 2015; subsequent assessment: 2017) from the Mexico City Representative Diabetes Survey^36^ and under the assumption of maintenance of physical activity and sugar-sweetened beverages consumption patterns over four years. Separate linear models were fitted for each dependent continuous variable (triglycerides, waist circumference, and hemoglobin A1c), using sugar intake from drinks and step counts as the primary predictors. The models were adjusted for age and sex. Fatty liver disease status was defined as a BMI at obesity levels (≥ 30 kg/m^2^) or a one-unit change between registries, in addition to triglycerides exceeding 200 mg/dL^37^.The higher the sugary drinks consumed and the lower the number of steps, the higher the indicator levels, and vice versa (i.e., also reminding users of beneficial changes), reflecting a "virtually healthy or sick" *Avatar Metabólico.* Our *Avatar* has four body image patterns: (1) normal weight; (2) overweight; (3) obesity; (4) morbid obesity.

### Complementary educational module

The complementary module of *Salud Activa* was *Componente educativo* (English; Educational component; Fig. 1F). The elements of this module were integrated by a graphic designer and health communication specialist to offer users attractive evidence-based guidance on the various biochemical markers related to NCD. Topics comprise (1) fatty liver disease; (2) hypertriglyceridemia; (3) hemoglobin A1C; (4) abdominal obesity; (5) BMI; and (6) physical activity. A web-based content manager was developed to modify the materials displayed on this application's component without programming.

### Pilot stage: application release to potential users

Inclusion criteria (stage of behavior change) were evaluated in 989 individuals; 520 (52.6%) were eligible and accepted to participate. Individuals were considered ineligible if they were identified at stages of behavior change other than contemplation or preparation. Of eligible participants, 515 continued until the end of the pilot stage. The average age of participants was 21.2 ± 4.6 years (24–45 years), and 63.1% were women. The median number of days participants logged into *Salud Activa* was seven (P25 = 3, P75 = 12; interquartile range = 9). Of the total study sample, 17.3% completed all questions on sociodemographic characteristics administered through *Encuestas*, and 35% responded to all questions related to NCD history. Among the respondents, 2.2% self-reported a medical diagnosis of diabetes, 2.2% of hypertension, 1.1% of fatty liver disease, 5% of hypertriglyceridemia, and 2.2% reported hypercholesterolemia. Physical activity questions were answered by 22.7% of participants, and 21.6% and 4.3% of them completed queries on consuming sugary drinks and sedentarism, respectively. Thirteen-point 6% of the study sample filled out at least one question related to blood pressure levels, glycemia, or lipid profile, 4.3% answered questions about the perception of obesity and body image, 6.2% about knowledge of food labeling, and 6.6% about depression. Regarding *Escáner nutrimental*, users registered 157 new food products on the platform. The information was validated and included in our database.

### Lessons learned and potential improvements

**Figure 3:**
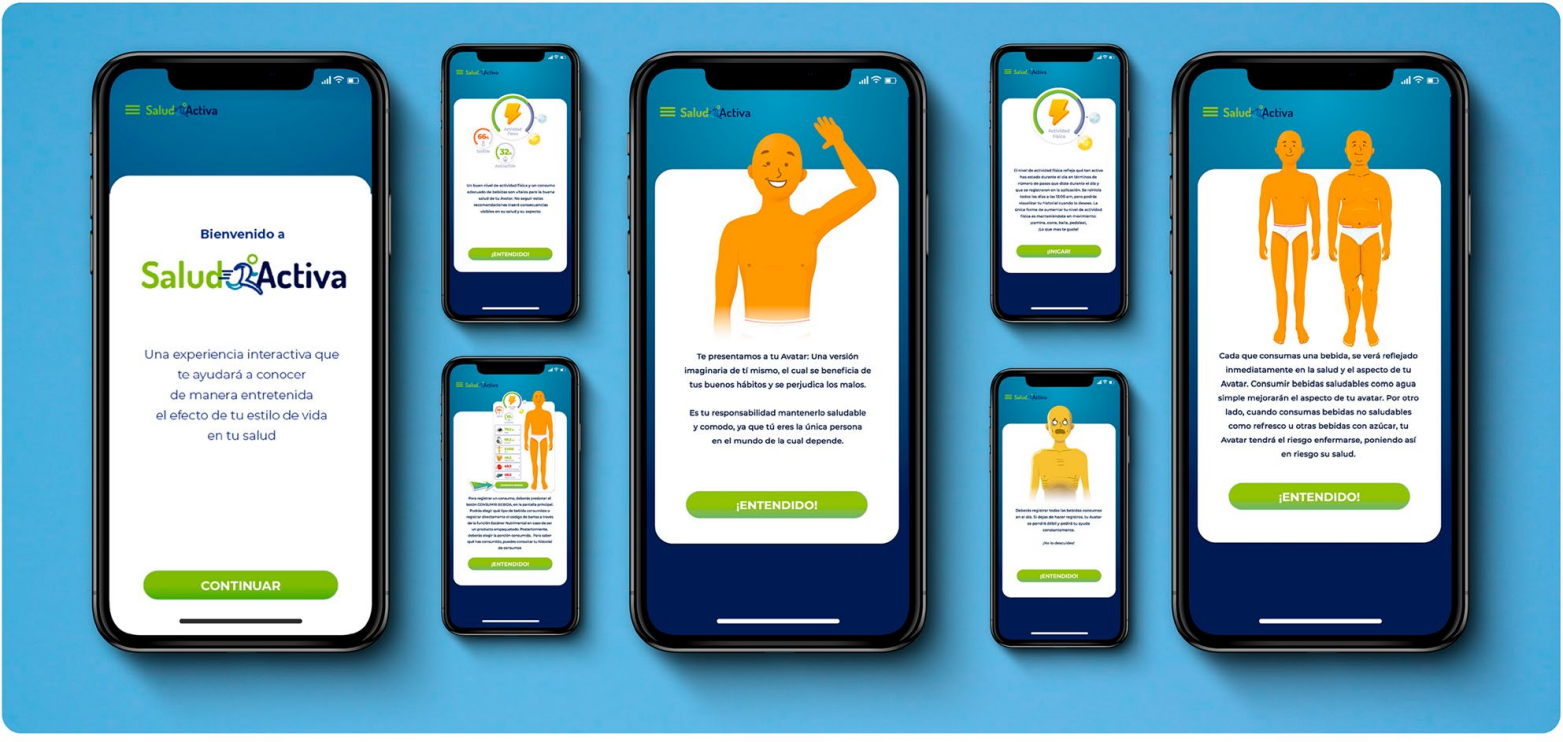
Redesign concept to improve *Salud Activa’*s functionality and user experience: new user guides, improvements in customization, and an enhanced Avatar. The figure depicts a new application design with an enhanced initial section with instructions on the interaction mechanics, objectives, and mission. The application mockups depict design enhancements to achieve more interesting, attractive, playful, logical, realistic, transparent, and tangible stimuli. The design aspires to appeal to empathy more than realism, seeking not to represent any ethnic, economic, cultural, or social group to reduce user rejection, and to maintain user retention through enhanced gamification dynamics and a personalized user experience.

Opportunities to improve *Salud Activa*'s quality of usability, transparency and content, technological components, and design were identified. First, the application's high content and interaction complexity could compromise users' interest and commitment. These challenges could be addressed by creating a more comprehensive initial section to guide the user on the application's interaction mechanics, objectives, and mission (Fig. 3).

**Figure 4:**
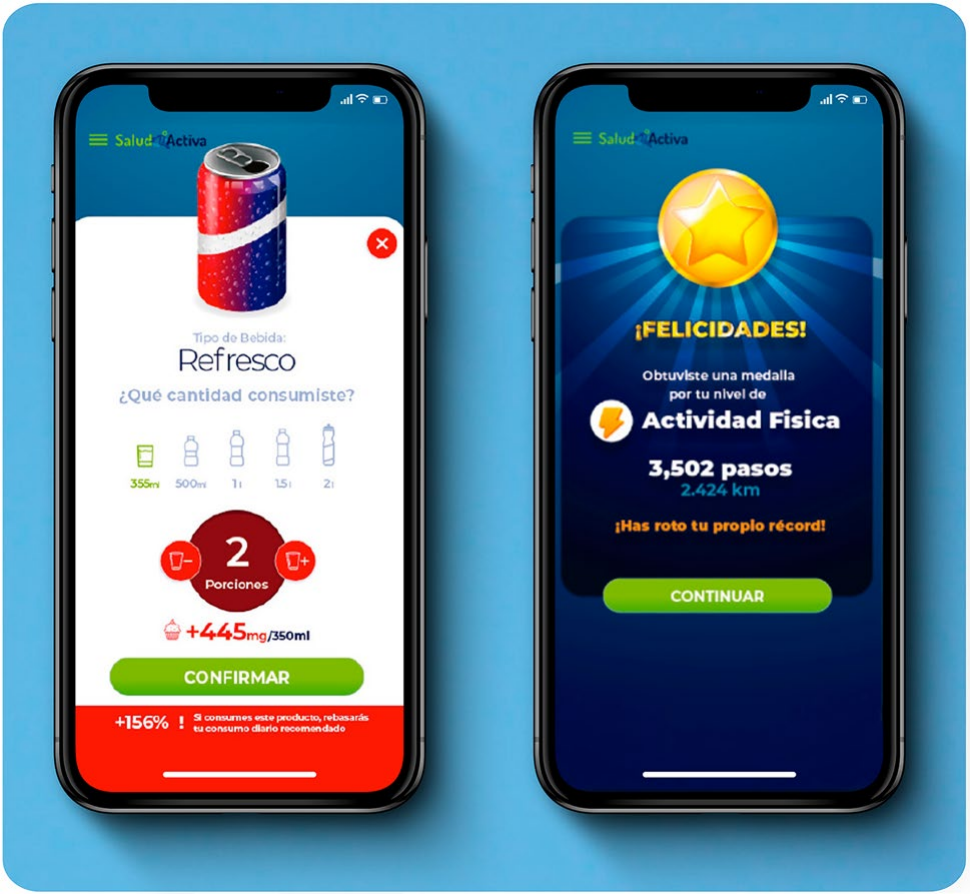
A new format of *Encuestas and Registro de bebidas*: enhanced design of crowdsourcing components of *Salud Activa.* The redesigned *Encuestas* interface (on the left) is more colorful, vibrant, visually engaging, and user-friendly. It includes graphical support on portion sizes, confirmatory touch buttons to ensure accurate registration, question grouping, control customization, fewer steps to complete items, and a function allowing the user to exit the question item without completing it. Furthermore, a warning message is designed to appear if the recommended intake of sugars is exceeded. The new style of indicators and rewards for *Contador de pasos* is shown on the right. Indicators and rewards, presented to users in a colorful and playful manner, are grounded in evidence-based goals. They include achievement rewards, historical level tracking, and record-breaking, all aimed at enhancing the personalized user experience.

Specifically, a new format of *Encuestas* and *Registro de bebidas* could increase response rates through question grouping, control customization, fewer steps to complete items, and allowing the user to exit the item without completing it (Fig. 4).

Improvements in the customization of *Salud Activa* and certain of its components could benefit long-lasting user engagement. These enhancements can involve more interesting and tangible stimuli, attractive and interactive graphics, and improved playful elements with more logical, realistic, and transparent processes. A new Avatar could appeal to empathy more than realism, seeking not to represent any ethnic, economic, cultural, or social group to reduce user rejection. The improved Avatar may also feature other interaction mechanics, such as fading after registering low water consumption to notify the user of the healthy hydration benefits (Fig. 3). While the theoretical design derived in the selection of step count and sugary drink intake as feasible targets in preventing ectopic fat accumulation, we acknowledge that weight status and other cardiometabolic markers result from a complex causal pathway. Hence, an improved Avatar should incorporate additional input variables triggering change in its indicators, such as the consumption of fruits, vegetables, nuts, whole grains, fish, and water. Critically, biomarker indicators (i.e., triglycerides, hemoglobin A1C, and waist circumference) integrated into the current Avatar may fluctuate based on drink registry or step count. Hence, it is crucial to include a specific cautionary alert within an enhanced Avatar to deter self-diagnosis, which could potentially result in adverse outcomes, such as erroneously bypassing a necessary medical evaluation.

A new style of indicators and rewards for *Contador de pasos* based on evidence-based goals, achievement rewards, historical level tracking, and record-breaking, considering other types of physical activity beyond steps (e.g., yoga or weightlifting), would likely also improve the personalized user experience (Fig. 4). It will be necessary to ensure automatic interaction between the smartphone's native pedometer and the application running in the background.

*Escáner nutrimental* is designed to exclusively monitor processed food items with a barcode, also leaving room for improvement. Enhancing the system to collect data on unprocessed/minimally processed fruits, vegetables, legumes, nuts, whole grains, fish, seafood, poultry, and water, and integrating a dynamic rewarding feedback mechanism would be a mutually beneficial strategy for both the research team and potential users. Another limitation we encountered was the oversight in incorporating a function to track participant count within *Escáner Nutrimental*. Monitoring this metric will be crucial for the future iterations of *Salud Activa*. It will allow the team to discern any disparity between the number of application users and the number of individuals actively contributing to this crowdsourcing initiative. With these data, our team could introduce personalized notifications or rewards to encourage greater engagement with food product registration through *Escáner Nutrimental* among users who show limited interest in this activity. Consequently, this would enhance the richness of our databases.

While the design process of the application was guided by the DIC, it was not possible to follow-through all the steps, due to time and economic constraints. For example, the formulation of the DIC-defined value proposition necessitates consideration of three key factors to ensure a net positive impact in potential interventions: alternatives, resources, and people's preferences^20^. Hence, we meticulously examined a range of alternative interventions varying in resource requirements that could potentially reduce sugar-sweetened beverage consumption and increase physical activity levels. For instance, traditional face-to-face nutritional counseling through digital platforms, existing smartphone applications with subscription fees or advertising, and the utilization of activity-tracking devices. Following thorough internal deliberation, we determined that an advertisement-free, open-access application compatible with popular smartphone platforms (iOS and Android), offering evidence-based information, would provide the greatest net value to the target population. Nonetheless, the formulation of the value proposition can be improved if one can assess the preferences and feedback of the target demographic through structured interviews and focus groups; this could be done in future iterations to refine the design of *Salud Activa* further.

## Discussion

The mHealth strategy herein created aspires to be a complementary digital instrument for preventing and controlling NCD utilizing open- and broad-access technologies in Mexico. *Salud Activa* was developed through Design Sprints^24^, considering the guidance of health- and nutrition-related experts and user needs. Scrum^25^ facilitated efficient time and resource management in this project.

mHealth strategies construction involves behavioral, computer, and physiology sciences. The likelihood of crafting an effective intervention is heightened when integrating theories of these fields and human–computer interaction knowledge^16–18^. Despite this understanding, uncertainties emerged in this project, particularly in how to operationalize scientific literature into technological products to promote healthy lifestyles and positive metabolic change for NCDs. To address these issues, we utilized a series of frameworks and evidence-based models around NCD pathophysiology^19^, dietary^20^ and physical activity^21^ interventions, and behavior change techniques supported by experts^22,23^. The creation of *Salud Activa* and the documentation of its theoretical and technological design processes may serve as a reference and incentivize the development and evaluation of other mHealth strategies in the realms of public health and healthy lifestyles in Mexico, where a paucity in the field is observed.

Three of the modules nested within *Salud Activa,* are intended to serve as platforms for delivering future behavioral change interventions aimed at preventing and controlling diabetes. These interventions specifically target positive changes in diet and physical activity. Their theoretical design is formulated with the objective of alleviating stress on pancreatic beta cells and mitigating ectopic fat accumulation^19^. The design process suggested that this physiological response may be achieved through a reduction in the consumption of sugary drinks, with a specific emphasis on minimizing fructose intake, and concurrently, by increasing the duration of moderate to vigorous physical activity. Among the various behavioral change techniques (e.g., fear appeal) considered to operationalize an intervention component with these features, we opted for education regarding the effects and onset time of the exposure and outcome. To ensure adherence, graphical and textual feedback and establishment of personalized goals regarding the desired healthy behaviors (e.g., optimal sugar intake or physical activity levels) were incorporated into the strategy. Regarding sugary drinks, the design process suggested monitoring the moments of beverage selection and intake per se. In terms of physical activity, it was recognized that encouraging an increase in the number of steps throughout the day is a viable intervention to achieve the objectives outlined in *Salud Activa.* To enhance sustained engagement with the intervention components, we reinforced the selected behavioral change techniques by integrating them into an avatar, a digital self-representation within a gamification strategy. This approach has been shown to be useful to transform experiences from unmotivating into enjoyable, offering a range of emotional and cognitive benefits in the process of acquiring knowledge, and improving satisfaction, self-esteem, and motivation among users^13,14^.

Using evidence-based elements, gamification was leveraged as a fundamental pillar to achieve durable user engagement, as observed in other eHealth strategies^13,38^. Crowdsourcing elements in this application represent a double-duty strategy. First, the potential for *Salud Activa* to complement the study of NCD dynamics in Mexico through participatory surveillance^39^, similar to what other mHealth platforms have done in large study samples from the Americas, such as the Saúde na Copa^38^ or Flu Near You^40^. Second, this application can generate nutritional awareness while monitoring food environment components in Mexico through its *Escáner nutrimental*. An example of these tasks is Food Switch, an application initially downloaded by 400,000 users gathering information on 30,000 products in the Australian food market^41^.

The beneficial essence of *Contador de pasos* and *Registro de bebidas* in *Salud Activa* has been utilized in other mHealth platforms by recognized organizations. For instance, in collaboration with the WHO and the American Heart Association, Google incorporated Heart Points into Google Fit to monitor physical activity and reward users for meeting evidence-based goals, an effective strategy to encourage active lifestyles^42^. Health (an Apple application) has been used to study changes in physical activity levels^43^. Applications for self-monitoring of dietary intake have also shown promising results. Two examples are Diet-A, which reduced sodium intake in adolescents^44^, and MealLogger, which increased athletes' nutrition knowledge^45^.

Digital representations that are responsive to user´s activities are often combined with gamification strategies in mobile applications ^13,13^. Some have successfully modified dietary and physical activity behaviors in adults^46,47^. Others provide valuable information for preventive interventions, such as Inside Me (Apple’s application), which can predict the probability of coronary heart disease and diabetes risk through data collection and statistical modeling involving interaction between users and their avatars^48^. Another application that links the users' activity to the status of an avatar also includes an online community that allows interaction between users^49^. This helpful functionality may increase engagement in interventions.

We did not identify Mexican studies allowing us to contrast basic data on the interaction level of our participants with our application versus others. However, the 3-month median of log-ins to *Salud Activa* was seven days, and up to 35% of participants completed an entire crowdsourcing-based questionnaire section embedded in the application. These are higher tendencies than the log-ins and user proportions using certain components in a similar platform tested in a randomized trial^50^. We found variability in ideal log-in frequencies yielding positive results. For example, an Australian 6-month intervention on step count involving a mobile application among university students and staff reported a mean usage of 15.7 days, resulting in statistically significant benefits^51^. Developers of a diabetes care application in Singapore found that recommending participants to log their weight once a week on the platform produced clinically relevant beneficial effects on diabetes control markers after a 24-week intervention^52^.

Of note, aiming to perform an early original design-based mobile application piloting, *Salud Activa* was released to a sample of potential users under uncontrolled conditions without any continuous stimulus to encourage further interaction. Similar applications have achieved high user retention and participation through regular push notifications (e.g., twice per week) or reminders through text messages for specific activities (e.g., assessments)^28,50,53^. Therefore, an intense, controlled study incorporating comparison groups in Mexico may elucidate which stimulation strategies (e.g., recruitment of groups with pre-established social ties or the creation of online communities) can augment the use of *Salud Activa* and the efficacy of its intervention components, namely *Avatar Metabólico, Registro de bebidas,* and *Contador de pasos*.

Lastly, it is worth noting that our project utilized data from iOS and Android applications, as well as smartphone's built-in features like pedometers. However, the integration of artificial intelligence (AI) in smartphone and mobile wearable devices and applications can significantly enhance health monitoring capabilities. This includes more accurate tracking of physical activity levels and heart rate, as well as the ability to detect mental health crises and falls more effectively^54^. *Salud Activa* was primarily developed using Application Programming Interfaces (APIs), which serve as critical components for integrating advanced technologies like machine learning AI. Hence, we propose that future mhealth strategies, akin to ours, integrate these tools to enhance both their development and evaluation processes^55^. For example, in Ghana, an application was tested to help users monitor their caloric intake and predict foods required to meet their energy requirements using AI^56^.

Contrast with other digital platforms shows that *Salud Activa*—mHealth strategy combining gamification and crowdsourcing elements—has the potential to promote healthy lifestyles and complement epidemiological studies in Mexico. A redesign plan for this application will emerge from a data triangulation analysis, including quantitative and qualitative information on its use. In parallel, the opinion of expert application developers with a background and proven results in health and nutrition areas will be considered. It is necessary to implement a comprehensive pilot test to evaluate usability metrics, including active users with segmented analysis, navigation flow and time spent within the application and its components, user retention, and indicators of interaction and engagement (e.g., through robust heat mapping). Direct interviews and focus groups would help identify users' technical difficulties, tastes, or preferences organically. This redesign plan is expected to enhance greater uptake, user retention, and lasting interaction with the application once *Salud Activa'*s areas of improvement are addressed.

## Data Availability

The data analyzed and presented in this study are available from the corresponding author on reasonable request.
